# Association between localized geohazards in West Texas and human activities, recognized by Sentinel-1A/B satellite radar imagery

**DOI:** 10.1038/s41598-018-23143-6

**Published:** 2018-03-16

**Authors:** Jin-Woo Kim, Zhong Lu

**Affiliations:** Roy M. Huffington Dept. of Earth Sciences, Southern Methodist University, Dallas, Texas USA

## Abstract

West Texas’ Permian Basin, consisting of ancient marine rocks, is underlain by water-soluble rocks and multiple oil-rich formations. In the region that is densely populated with oil producing facilities, many localized geohazards, such as ground subsidence and micro-earthquakes, have gone unnoticed. Here we identify the localized geohazards in West Texas, using the satellite radar interferometry from newly launched radar satellites that provide radar images freely to public for the first time, and probe the causal mechanisms of ground deformation, encompassing oil/gas production activities and subsurface geological characteristics. Based on our observations and analyses, human activities of fluid (saltwater, CO_2_) injection for stimulation of hydrocarbon production, salt dissolution in abandoned oil facilities, and hydrocarbon extraction each have negative impacts on the ground surface and infrastructures, including possible induced seismicity. Proactive continuous and detailed monitoring of ground deformation from space over the currently operating and the previously operated oil/gas production facilities, as demonstrated by this research, is essential to securing the safety of humanity, preserving property, and sustaining the growth of the hydrocarbon production industry.

## Introduction

Geohazards pose a severe threat to humanity, civilian properties, infrastructures, and industries, possibly leading to the loss of life and high economic values^1^. Monitoring areas prone to geohazards is invaluable for locating their precursory signals on the surface, alerting civilians to potential disasters, mitigating the catastrophic outcomes, and facilitating the decision-making processes on the construction and operation of infrastructures and industrial facilities. The United States mid-continent has long been considered geologically stable with no large scale tectonic movements, volcanism, or seismic activities^2,3^. Therefore, unlike California with its dense GPS networks and frequent survey (aerial, spaceborne, field) campaigns, the mid-continent has garnered less attention from scientific communities and federal/state governments. However, recent studies have revealed that some of the mid-continent, especially the Gulf Coast of the United States including Texas, Louisiana, and Mississippi, is not immune to large-scale and/or localized geohazards^4,5^.

The geohazards along the southern United States have been both naturally induced and stimulated by human activities^1,3^. Besides the occasional, strong tropical storms and flooding in lowlands, natural geohazards include settlement due to sediment loading and glacial isostatic adjustment, which can make the coastline in the Gulf Coast vulnerable to sea-level changes^6–8^. However, the naturally occurring surface subsidence on the coast displays characteristics of a continuous, slow progression (millimeters per year) and a large spatial extent (~100 km wide)^6^. In contrast, human-induced geohazards are faster growing (up to tens of cm/yr) and encompass a varying but generally small area (up to a couple of km wide). The most prominent difference between natural and human-induced geohazards is the correlation between surface instability and anthropogenic activities (e.g., mining, groundwater extraction, hydrocarbon production)^3,9^. Although there can be a time delay of ground deformation after human activities, depending on the geological characteristics (porosity, elasticity, compressibility, pore pressure, permeability) of soils and rocks and types of the operations, human-induced surface subsidence or uplift usually has high proximal and temporal correlation with those activities^10–12^.

West Texas is somewhat distant from the Gulf coast, but was inundated by relatively shallow seas during the early part of the Paleozoic Era (approximately 600 to 350 million years ago). The sediments formed during this period contributed to the accumulation of sandstone, shale, and limestone. The seas constituting broad marine environments in West Texas gradually withdrew, and by the Permian Period (approximately 299 to 251 million years ago), thick evaporites (salt, gypsum) accumulated in a hot arid land encompassing shallow basins and wide tidal flats. As a consequence of geological formation in West Texas, the deposited carbonate (reef limestone) and marine evaporite sequences played an important role in the formation of oil reservoirs by helping seal the traps and preserving the hydrocarbons^13^. This resulted in the Permian Basin of West Texas’ massive hydrocarbon reservoirs that became so lucrative to the oil and gas industry^14^.

In West Texas, human activities such as groundwater exploitation, fluid injection, and hydrocarbon extraction have resulted in surface instability, leading to geohazards such as surface heave/subsidence, fault reactivation^4^, induced seismicity^15,16^, and sinkhole formation^17–19^. The vastness of West Texas challenges our ability to identify and locate the relatively small spatial scale of the deformation corresponding to human activities, particularly for fluctuations over the course of a month or a year. Without concerted focus, the small-sized signal in a short time window can go easily undetected. There have been a few studies documenting the surge of surface uplift/subsidence, sinkhole formations, and induced seismicity in oil fields^19–22^. However, the role of human activities on the surface and subsurface deformation has yet to be fully established, particularly regarding the identification of small-scale deformation signals over a vast region from big datasets spanning multiple years and analyzing them with supplementary information.

**Figure 1:**
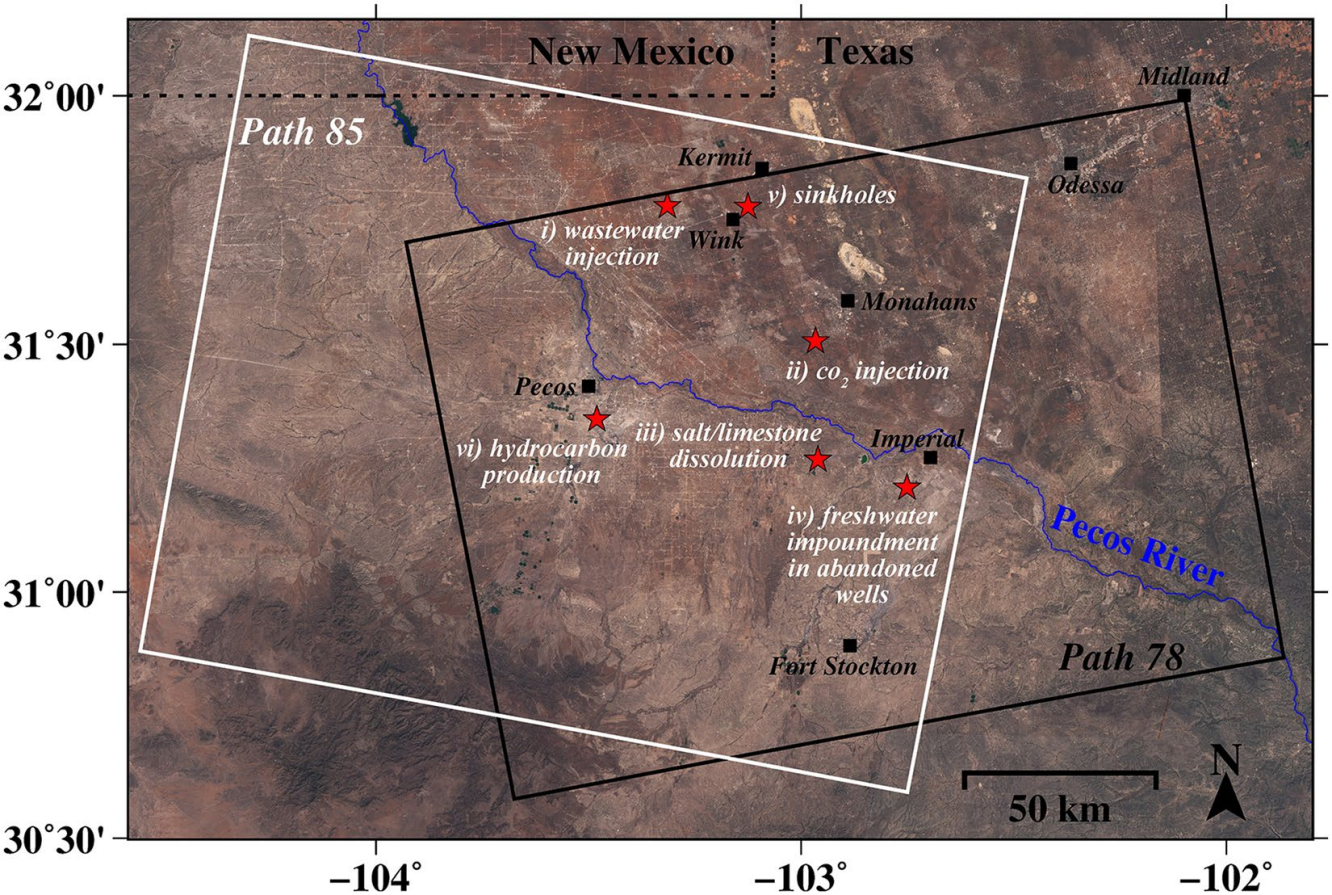
Locations of ground deformation in West Texas. 6 major sites (red stars) in West Texas display the locations influenced by human activities identified based on Sentinel-1A/B multi-temporal interferometry (background image is from Sentinel-2). To estimate 2D (east-west and vertical) deformation, the ascending (path 78; black box) and descending (path 85; white box) track Sentinel-1A/B images were integrated over the overlapped regions. West Texas’ Permian Basin contains two major aquifer systems under the influence of the Pecos River, the Pecos Valley aquifer and the Edwards-Trinity aquifer. The figure has been created using open-source software Generic Mapping Tools (GMT) 5.2.2_r15292 available at http://gmt.soest.hawaii.edu/projects/gmt/wiki/Download. The Sentinel-1A/B data used in this study were downloaded in 2017 through the Vertex online archive https://vertex.daac.asf.alaska.edu provided by Alaska Satellite Facility (ASF) and the Sentinel-2 data used as a background image for this figure were obtained in 2017 through Copernicus open access hub https://scihub.copernicus.eu provided by the European Space Agency (ESA)’s Copernicus Programme.

Challenges to the effective study of the geohazards in West Texas include: identification of their locations in remote and vast regions, measurement of their long-term evolution, and characterization of the causal mechanism with accessible information. Satellite radar interferometry (InSAR) has proven capable of imaging ground surface deformation with a measurement accuracy of centimeters or better at a spatial resolution of meters or better over a large region covering tens of thousands km^2,23^. However, satellite radar acquisitions over West Texas have previously been scarce. Here we present the analysis of the ongoing ground deformations induced by various geohazards around Pecos, Monahans, Wink, and Kermit in West Texas (Fig. 1), using multi-temporal InSAR observations based on radar imagery from the first free, open-source radar satellites Sentinel-1A/B. The objective of our study is to probe the association between the ongoing localized geohazards in West Texas and anthropogenic activities. To achieve the goal, we focus on the localized, small-sized (200 m~2 km wide), and rapidly developing (cm/yr) geohazards in the region, which are categorized based on six possible causes: i) wastewater injection, ii) CO_2_ injection for enhanced oil recovery (EOR), iii) salt/limestone dissolution, iv) freshwater impoundment in abandoned wells, v) sinkhole formation in salt beds, and vi) hydrocarbon production. In addition, time-series measurements from two different imaging geometries are integrated to decipher the deformation phenomena. Furthermore, through comparative analysis of records of fluid injection, hydrocarbon production, and geological characteristics, we establish the relationship between the possible causes of human activities or natural perturbation and the localized observed geohazards in West Texas.

## Results

Here we report local geohazards occurring in West Texas, most of which have not been noticed and reported yet. Knowledge of the presence of the ongoing geohazards in West Texas is a precursor to understanding the trigger and causality of ground deformation, the revelation of which is a focal point of our study. The localized geohazards presented below may have different characteristics in spatio-temporal progression and causality (i.e. wastewater injection, CO_2_ flooding, hydraulic fracturing, freshwater impoundment), but all are happening because West Texas contains a sequence of water-soluble (limestone, evaporite) and shale formations that are highly vulnerable to human activities.

### Wastewater injection into formation and surface uplift

**Figure 2:**
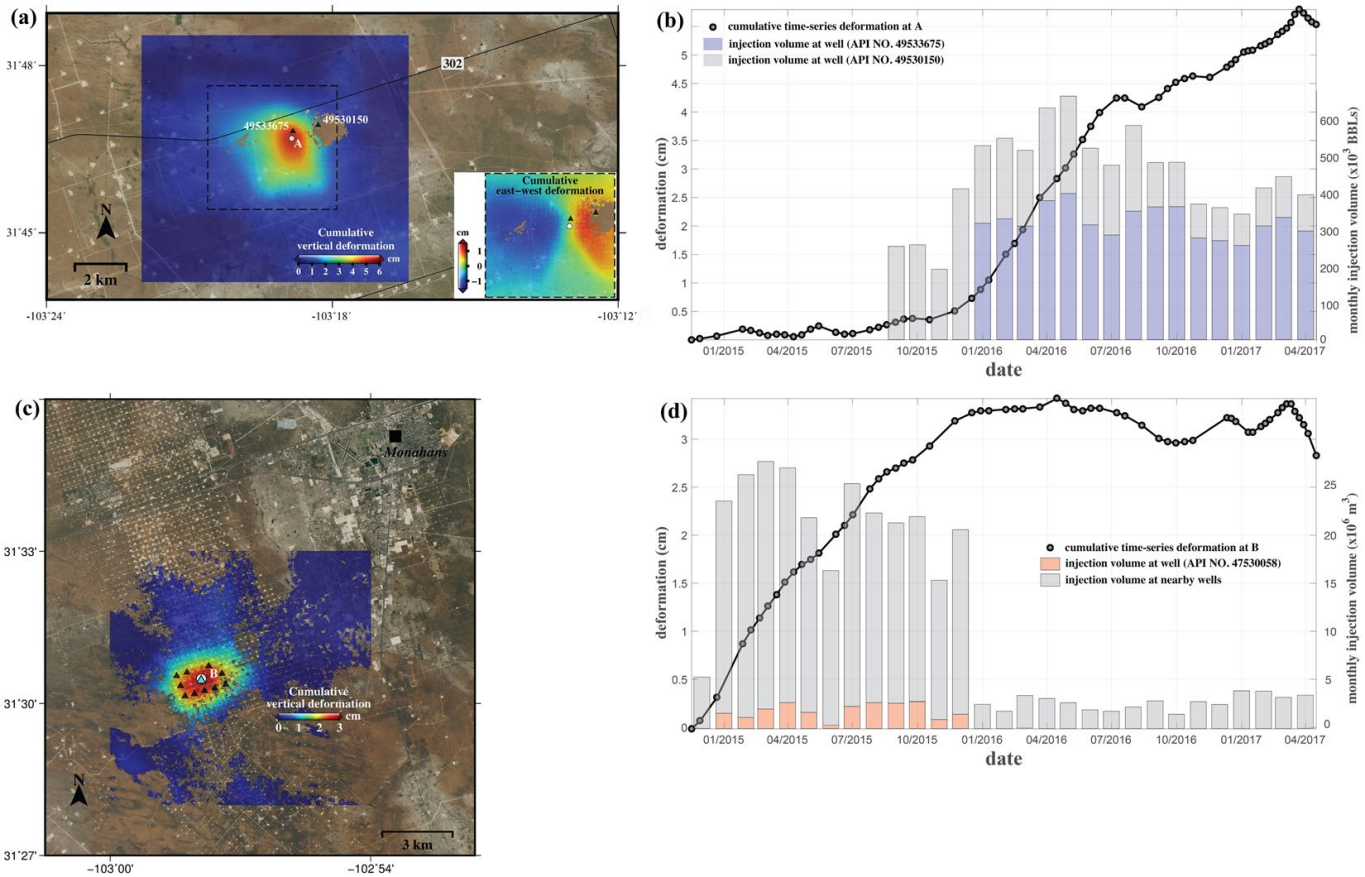
Ground uplift due to fluid (wastewater, CO_2_) injection. (**a**) Uplift in Winker County, TX, induced by wastewater injection in nearby wells (API No. 49533675, 49530150). Inset illustrates cumulative east-west deformation in the box outlined by a dashed rectangle. (**b**) Time-series cumulative vertical deformation in a point A (Fig. 2a) and the volume of injected wastewater (blue and gray bars) in two injection wells. (**c**) Uplift in Ward County, TX, induced by CO_2_ injection in an EOR field (triangles). (**d**) Time-series cumulative vertical deformation in a point B of Fig. 2c and the volume of injected CO_2_ (orange and gray bars) in EOR injection wells of Fig. 2c. The figures including spatial information have been created using open-source software GMT 5.2.2_r15292 available at http://gmt.soest.hawaii.edu/projects/gmt/wiki/Download. The National Agriculture Imagery Program (NAIP) images used as a background of the figures were downloaded in 2017 through Geospatial Data Gateway https://datagateway.nrcs.usda.gov provided by United States Department of Agriculture (USDA).

Wastewater ‘flowback fluid’, a byproduct of oil and gas production^24^, has been injected deep underground about 15 km west of Wink and Kermit, Texas (Fig. 1). The hydrocarbon production in the Bone Springs reservoir requires hydraulic fracturing, and wastewater (also called brine) containing high concentrations of total dissolved solids (TDS) is produced as a result of the operations. Two wells (API No. 49533675 and 49530150 in Fig. 2a) located near the county border between Winkler and Loving counties are classified as Class II injection wells for disposal of saltwater and non-hazardous fluids into the subsurface as a result of oil and gas production. The injection depth is from 1,590 to 1,670 m where the Bell Canyon Formation in the Delaware Basin of the larger Permian Basin lies. The upper layer (~10 m thick) of the formation is composed of limestone that can confine the upward flow of injected fluids. Most wastewater is injected below the nearly impervious limestone units, into the Bell Canyon Formation sandstones (also called Ramsey sandstones); these sandstones have a porosity of ~20% of open pore-space for holding fluids, and a moderate-to-low permeability (a measure of how readily fluids can flow through the rock) of ~40 md (millidarcy)^25^. Our InSAR analysis has detected the surface upheaval approximately centered on the injection well No. 49533675 (Fig. 2a). The maximum (cumulative) uplift from late 2014 to April 2017 was ~5.5 cm with the shape of a distorted ellipse, and the influential zone is within a 2 km radius of the peak deformation (white dot labeled ‘point A’). Horizontal (east-west) deformation with the maximum of ~1.2 cm is also occurring on both the east and west sides of the peak uplift (inset in Fig. 2a), with the western region moving to the west (negative, blue color) and the eastern region moving to the east (positive, red color). The horizontal deformation around the injection wells represents <~20% of the vertical (up-down) deformation; we therefore concentrate on the vertical deformation in this study.

Generally, surface uplift can be caused by the expansion of the geological formation where the fluid is injected, resulting in the upward movement of the ground surface. The injected formation experiences an increase in pore pressure as well as a decrease of the effective stress^26^, promoting the surface uplift as we observed^27^. At the point of maximum uplift (A in Fig. 2a), uplift was detected beginning around September 2015, with a sharp increase (at a rate of ~6 cm/yr) during the first half of 2016 (Fig. 2b), and the value after October 2016 sustained near ~5 cm cumulative deformation in spite of some monthly fluctuations. The temporal changes in uplift seem to be in concert with the changes in injection volume, suggesting that a mechanical compaction of sands by means of poroelasticity is likely the primary cause of the deformation^28^. In addition, the small variations in the vertical deformation since mid-2016 can be depicted as the combined effects of poroelastic compaction and viscoelastic behavior of fine-grained formations surrounding an injected strata^28,29^. We can also infer that, relatively speaking, the upper (sandy) layer responds rapidly to waste water injection, but the lower (shale/silt) formation reacts gradually to changes in overlying stress.

To unravel the causality of the surface uplift, we compared our deformation observations to the sequence of wastewater injection rates in the nearby wells. Based on the H-10 forms provided by the Texas Railroad Commission (RRC)^24^, the No. 49533675 injection well has been active since January 2016; the No. 49530150 injection well, which first became operational in 2009, experienced a period of disuse, and was then reactivated in September 2015 (Fig. 2b). The ratio of the uplift volume (i.e. the multiplication of the uplift amplitude and area extent) and the injection volume is about 0.05 m^3^/BBL (1 BBL ≈ 0.12 m^3^). Based on the onset of the uplift coinciding with the reactivation of 49530150 in September 2015, along with the increasing uplift rate aligning with the use of 49533675, it seems likely that both injection wells were affecting the surface uplift, but the effects of the two are not equivalent. It seems that the injection well No. 49533675, closest to the peak of vertical deformation, is likely situated in a geologically weak, critical formation, allowing the injection/disposal of wastewater to influence the surface deformation more dominantly. The correlation between the vertical deformation and the wastewater injection suggests that the expansion of injected formations induced a localized, relatively small-sized (~2 km in dimension), and small-magnitude (~5 cm) surface uplift. Although the onset of ground uplift was most likely triggered by the wastewater injection and the nearly instantaneous response of the ground surface results from the high elasticity of the underlying formations, the correlation between wastewater injection and ground surface may not be as high as we expect. The stratigraphic response to the decreased or increased pore pressure and effective stress can be a complicated process. When the injected volume is in decline, the release of relative pore pressure allows for the different response, namely immediate downward movement of coarse-grained formations and lagged upward movements of fine-grained formations. Such combined effects of the subsurface/surface processes result in retention of the intermediate correlation between the injection volume and ground uplift. The deformation has not invoked seismicity yet, but, if the injection continues, it has potential to threaten the integrity of County road 302, nearby oil/gas pipelines, and hydrocarbon production facilities.

### Carbon dioxide (CO_2_) injection and surface uplift

Miscible CO_2_ flooding has been applied for decades as a tool for EOR in the depleted oil and gas reservoirs of the United States^30,31^. Unlike water and oil, supercritical fluid CO_2_ and oil mix together well, forming a single homogenous, or ‘miscible’, fluid (CO_2_ has different properties, depending upon its physical state; At room temperature, CO_2_ is a gas, such as what we exhale; we use liquid CO_2_ as a coolant, and we refer to the solid form of CO_2_ as “dry ice”. Supercritical CO_2_, achieved under specific pressure and temperature conditions, is between gas and a liquid state, with some properties of each). CO_2_ is injected into a disposal well within a reservoir after initial hydrocarbon production rates have declined, where the CO_2_ mixes with the hydrocarbons. The CO_2_ injection causes an increase in reservoir pressure, which forces the CO_2_-oil mixture out of the pores of the rock and towards one or more producing wells, allowing more oil to be recovered from the reservoir. CO_2_ injection for EOR has been economically efficient due to its low cost, aiding the Permian Basin’s EOR boom in the 1970s and 1980s and the recent years^32,33^.

The North Ward Estes Field west of Monahans, Texas and near Wickett, Texas is one of the largest cumulative oil-producing field in the Permian Basin^34^ (Fig. 2c). The oil and gas are produced from the Yates and Queen Formations, within the Midland Basin of the larger Permian Basin. In CO_2_ (Class II) injection wells 11 km southwest of Monahans, Texas (Fig. 1), the CO_2_ is injected into both formations at depths between 750 and 810 m. Crude oil and gas can be produced from both Yates and Queen formations, but Yates consists of very-fine-grained sandstones to siltstones separated by dense dolomite beds and contains ~16% of porosity and ~37 md of permeability, providing more dominant production volume in the North Ward Estes Field^33^. Salt water injection is also used for EOR (either ‘water flooding’ by itself, or in alternation with CO_2_ flooding), but its use today in this region is very limited (1% of total injected fluids) and most injection for EOR relies on the miscibility of anthropogenic CO_2_ (99% of the total injected fluids).

Our multi-temporal InSAR analysis has detected the ellipse-shaped surface uplift (major and minor axis: 6 km and 4 km, respectively) in the immediate vicinity of the CO_2_ injection sites (Fig. 2c), with a cumulative uplift of ~3 cm from late 2014 to April 2017. At the point of maximum uplift (B in Fig. 2c), the cumulative uplift increased linearly (at a rate of ~3 cm/yr) until January 2016 after which the value stayed at ~3 cm cumulative uplift (Fig. 2d).

Within 500 m of the maximum uplift, 11 CO_2_ injection wells (triangles in Fig. 2c) remain active as of April 2017. Although there has been variation in the injected volume (gray bars in Fig. 2d), most injections of CO_2_ occurred during 2015, with much lower injection volumes (below 5 million m^3^) since January of 2016 (Fig. 2d). The API No. 47530058 injection well (cyan triangle in Fig. 2c; orange bars in Fig. 2d) lies in approximately the same location as the maximum uplift.

The mechanism of the surface uplift caused by the CO_2_ injection is almost identical to the wastewater injection-induced uplift. Injected fluids, in this case, liquid supercritical fluid CO_2_, increases the pore pressure in the rocks (sandstones in Yates Formation for the CO_2_ EOR sites) and the release of the effective stress is followed by surface uplift^26,27^. The fluctuations in deformation after the injection was slowed down or stalled can be due to the collective effects of poroelastic compaction and viscoelastic delayed uplift in the formations surrounding the injected layer^28,29^. The proximity between maximum uplift and the No. 47530058 injection well, implies that the CO_2_ flooding in that particular well is more influential on the movement of ground surface than other surrounding wells (black triangles in Fig. 2c).

The high correlation between a large amount of uplift (~3cm) and CO_2_ flooding during 2015 suggests that the instability of ground surface in the southwest of the North Ward Estes Field was induced by the pressurized injection of CO_2_ into the Yates formation. Contrary to the surface uplift in the vicinity of No. 47530058, no significant deformation has been detected on the other portions of the North Ward Estes Field. Differences in rock strength, porosity, compressibility, and permeability can play a role in the occurrence of deformation^28^. CO_2_ flooding has revitalized, and continues to enhance recovery of the mature oil fields of the Permian Basin, helping to produce significant volumes of oil without CO_2_ emission^32^. However, pressurized injection into a geologically unstable rock formation can destabilize the ground surface and risks the productivity of further oil operations^35,36^.

### Dissolution of salt/limestone in Santa Rosa Spring

**Figure 3:**
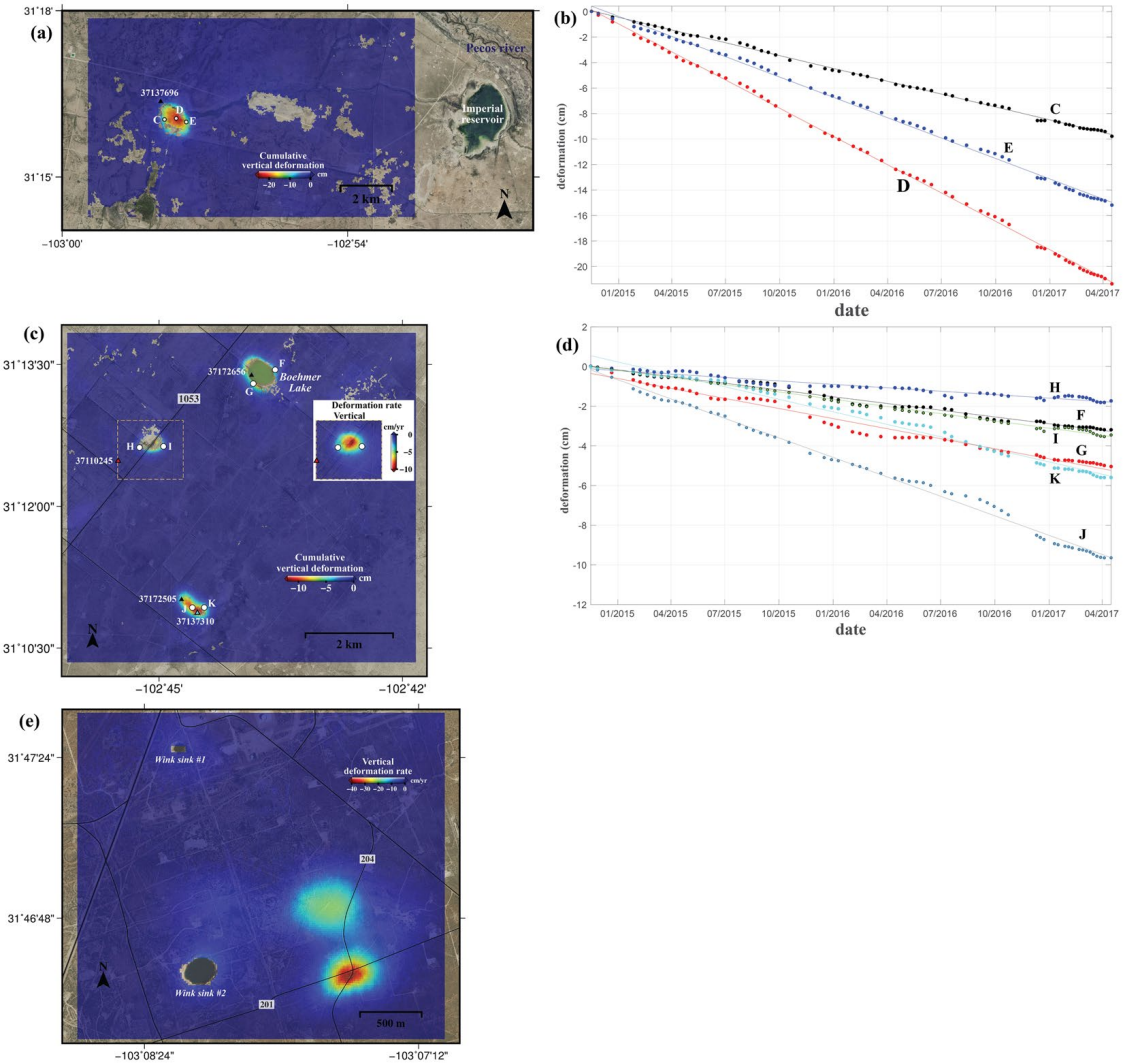
Ground subsidence in karst terrain underlain by limestone and salt. (**a**) Cumulative vertical deformation in Santa Rosa Spring. (**b**) Time-series cumulative vertical deformation at C, D, and E points of Fig. 3a. (**c**) Cumulative vertical deformation around abandoned wells in Imperial, Texas. Inset represents the averaged deformation rate in a boxed region by stacking interferograms of less than 12 days. (**d**) Time-series cumulative vertical deformation at F, G, H, I, J, and K of Fig. 3c. (**e**) Vertical deformation rate around Wink sinkholes. The figures including spatial information have been created using open-source software GMT 5.2.2_r15292 available at http://gmt.soest.hawaii.edu/projects/gmt/wiki/Download. The NAIP images used as a background of the figures were downloaded in 2017 through Geospatial Data Gateway https://datagateway.nrcs.usda.gov provided by USDA.

The Pecos County Water Improvement District No. 2 owns and operates a 2 km wide reservoir, known as the Imperial Reservoir (Fig. 3a), located about 6.4 km south of Grandfalls, Texas. Used for both irrigation of agricultural fields in Coyanosa, Texas and for recreational purposes, the Imperial Reservoir’s water is pumped from the Pecos River. In addition to the pumped Pecos River water, the reservoir also receives artesian spring water from the Santa Rosa Spring, 13 km southwest of Grandfalls, Texas in Pecos County, through narrow canals and channels.

Our InSAR analysis has detected rapid subsidence occurring in Santa Rosa Spring (Fig. 3a) from late 2014 to April 2017, with a maximum cumulative subsidence of approximately 23 cm, or at a rate of ~8.9 cm/yr (point D in Fig. 3a). The subsiding region is elliptical in shape with dimensions of ~1.4 km by ~1.0 km. Time-series deformation measurements at three points (C, D, and E in Fig. 3a) show a strong linearity (Fig. 3b), regardless of other factors of seasonal effects and irrigational uses.

Around the Santa Rosa Spring, hydrogen sulfide has been produced from multiple wells. However, the hydrogen production can hardly be directly connected to the rapid subsidence because all of the wells are located outside of the deforming zone that is centered on the Santa Rosa Spring. Hence, the hydrogen production should not have apparent impact on the observed subsidence.

Historically, a limestone cavern formed around Santa Rosa Spring, and the runoff water occasionally flows from and into the cavern^37^. Stratigraphical data for the area’s closest well, API No. 37137696 well (Fig. 3a), indicates Bone Springs Limestone formation is present at depth between 2,065 m and 2,911 m. However, the dissolution of this deep-seated limestone formation and the connection to the surface subsidence is not realistic as the extent of the subsidence area is less than 1.5 km (Fig. 3a). In addition, the dissolution rate of carbonate rocks like limestone is generally much smaller than that of the evaporite rocks, and a limestone cavern in a natural state forms very slowly (mm/yr)^38,39^. Therefore, such rapid subsidence rate (8~9 cm/yr) at Santa Rosa Spring is less likely caused by the natural dissolution of limestone.

Because of the nature of the rapidity in subsidence, we believe the most likely cause of the observed subsidence is the dissolution of the Salado formation in the depth of 300~450 m beneath Permian Basin. Investigations of groundwater conditions in Pecos County indicated that the highest salinity over the region was found at Santa Rosa Spring (7224 mg/l)^40^. In addition, it has been documented that the salinity over the Santa Rosa Spring increased by 4,894 mg/l from the 1940s to 1987^40^. Therefore, we interpret the rapid subsidence in the Santa Rosa Spring area to be caused by the dissolution of salt deposits. Although the subsidence has not triggered the collapse of the surface, the continuous surface subsidence in the areas can be hazardous to water management facilities and/or nearby oil/gas wells.

### Freshwater impoundment in abandoned wells

The region near Imperial, Texas (Fig. 1), has been troubled with the growing subsidence, ground fissures, and the emergence of sinking lakes^41,42^. Some abandoned water and oil wells were left unplugged and thus did not prevent freshwater impoundment through cracks in cement casing and/or the corroded steel pipes, and the freshwater impoundment is known to be the primary cause of rapid subsidence in the area^41^. However, prior to this study, subsidence near many abandoned wells has gone unnoticed^42^, and the Texas Department of Transportation is expected to spend millions of dollars to identify and plug the abandoned wells^41,42^. Our InSAR analysis has detected rapid subsidence around 7 km southwest of Imperial, Texas (Figs 1 and 3c). The region around Boehmer Lake (F and G in Fig. 3c) has sunk as much as 2~3 cm over the course of our InSAR acquisition period (2.5 years). Boehmer Lake did not exist before 2003 and the sinking ground surface led to the formation of the lake as a result of water arising from the subsurface, thus this is continued subsidence. Farm to Market (FM) road 1053 (near H, I in Fig. 3c) is sinking so fast that we could only compare pairs of satellite data within 12 days in order to maintain coherence of the InSAR image (inset in Fig. 3c). Therefore, using InSAR pairs with small (6 or 12 days) temporal baselines, we were able to measure the round-shaped (500 m in diameter) subsidence rate (~10 cm/yr) along FM 1053 road. Due to the safety concern, use of the road was suspended in August of 2016 and the realignment of FM 1053 was discussed by the state transportation agency^41,42^. A third nearby area of rapid subsidence (~10 cm/yr for 2.5 years) (near J, K in Fig. 3c) was observed near oil well API No. 37137310 (cyan triangle in Fig. 3c, near J and K). The subsidence pattern (650x350 m in dimension) is stretched NW-SE, aligning with two wells: API No. 37172505 and 37137310. Like the subsidence over the Santa Rosa Spring, vertical deformation measurements at points (F, G, H, I, J, K in Fig. 3c) show a strong linearity in time (Fig. 3d). Two points (F, G in Fig. 3c) in Boehmer Lake are experiencing 1.4 and 2.0 cm/yr subsidence. Points (H, I in Fig. 3c) near the outer edge of the deformation in FM 1053 road show the subsidence of 0.7 and 1.5 cm/yr, respectively. The areas near two oil wells are also undergoing subsidence of as much as 3.9 and 2.5 cm/yr, respectively. A few oil wells to the south of Imperial, Texas are currently active (i.e., No. 37137310 in Fig. 3c), with moderate production (less than 400 BBLs/month) for most of the time.

The observed linear subsidence relatively independent of oil/gas production and seasonal effects has the characteristics of ground subsidence (subsidence sinkhole) in karst terrain^19^. High salinity of channels along the Pecos River near Imperial, Texas and Boehmer Lake^43,44^ suggests that the surface and underground water interact with the subsurface salt deposit. The deforming area (Fig. 3c) is located in the Central Basin Platform close to the eastern Delaware Basin of the larger Permian Basin and is underlain by the Salado Formation in the depth of 300~500 m. Through unplugged abandoned wells, corroded pipes, or cracks in the casing, freshwater flows down and/or artisan water rises to the Salado formation, accelerating the dissolution of the evaporite, creating voids in the beds, and causing rapid subsidence on the surface^17,39^. Indeed, all three areas of subsidence are near wells. Boehmer Lake formed over an abandoned oil well (No. 37172656), which had stopped producing decades ago, and the subsidence along FM 1053 road is occurring near an orphan well (red triangle in Fig. 3c) that was identified as an inactive, non-compliant well by Texas’ petroleum regulatory agency (RRC)^45^. The oil production in No. 37137310 or related operations may influence the large rates of subsidence. However, the downward flow of freshwater into an unplugged oil well (i.e. No. 37172505) may play a more influential role in subsidence as the subsiding areas are all underlain by salt deposits. The dissolution of salt beds (evaporite) is typically more substantial than that of the carbonate rocks (limestone), and often exceeds ~10 cm/yr subsidence^38,39^. Expansion of the cavity and the migration of voids toward the surface can possibly result in the collapse of the surface into sinkholes. Therefore, movements around the roads and oil facilities to the southwest of Imperial, Texas should be thoroughly monitored to mitigate potential catastrophes.

### Dissolution of the salt bed near the Wink Sinkholes

Ground subsidence is more widely recognized near two Wink sinkholes, which collapsed in 1980 (Wink Sink #1 in Fig. 3e) and 2002 (Wink Sink #2 in Fig. 3e)^18,19^. The sinkholes, 4 km northeast of Wink, Texas and 9.5 km southwest of Kermit, Texas (Fig. 1), lie in the Delaware Basin part of the larger Permian Basin. The Salado Formation is near a depth of ~500 m^46^ over this area. The oil and gas in the region are mostly produced from the Yates Formation underneath the Salado Formation^19,46^. Both Wink sinkholes collapsed because of downward freshwater seeping through unplugged boreholes and cracked cement casing in oil and water wells. The subsidence in the immediate vicinity of the collapsed sinkholes continues at a rate of ~3–4 cm/yr (Fig. 3e).

The most significant ongoing subsidence is occurring 1 km east of the Wink #2. There are two large subsidence bowls (Fig. 3e), and the maximum subsidence in the southern bowl (380 m by 280 m in dimension) exceeds 40 cm/yr. The large gradient of subsidence in a small region cannot be observed by C-band InSAR pairs with 24-days or longer. Accordingly, only 5 InSAR pairs with 6 or 12-days temporal baselines are used to calculate the high linear deformation rate here. The peak subsidence is located at the intersection of County roads 201 and 204, and there are no existing active wells around the region. Therefore, the rapid subsidence is likely induced by the freshwater impoundments from the nearby abandoned wells. During our field trip, we observed numerous recent ground fissures around the intersection of County roads 201 and 204. These growing fissures can allow the rainwater to swiftly flow down to the Salado formation and promote the dissolution of the salt layers. Because the oil and gas production in the area has been inactive for years, the mechanism for both bowls is believed to be the same. The access to the region surrounding Wink #1 and #2 has been restricted out of safety concerns, but County Road 201 continues to be used to transport oil and gas products. Based on the observed rapid subsidence, the use of County Road 201 should be proactively monitored for safety. Additionally, the effect of ongoing subsidence on the pipelines in the area needs to be reviewed as well.

### Hydrocarbon production in Pecos and the associated seismic events

**Figure 4:**
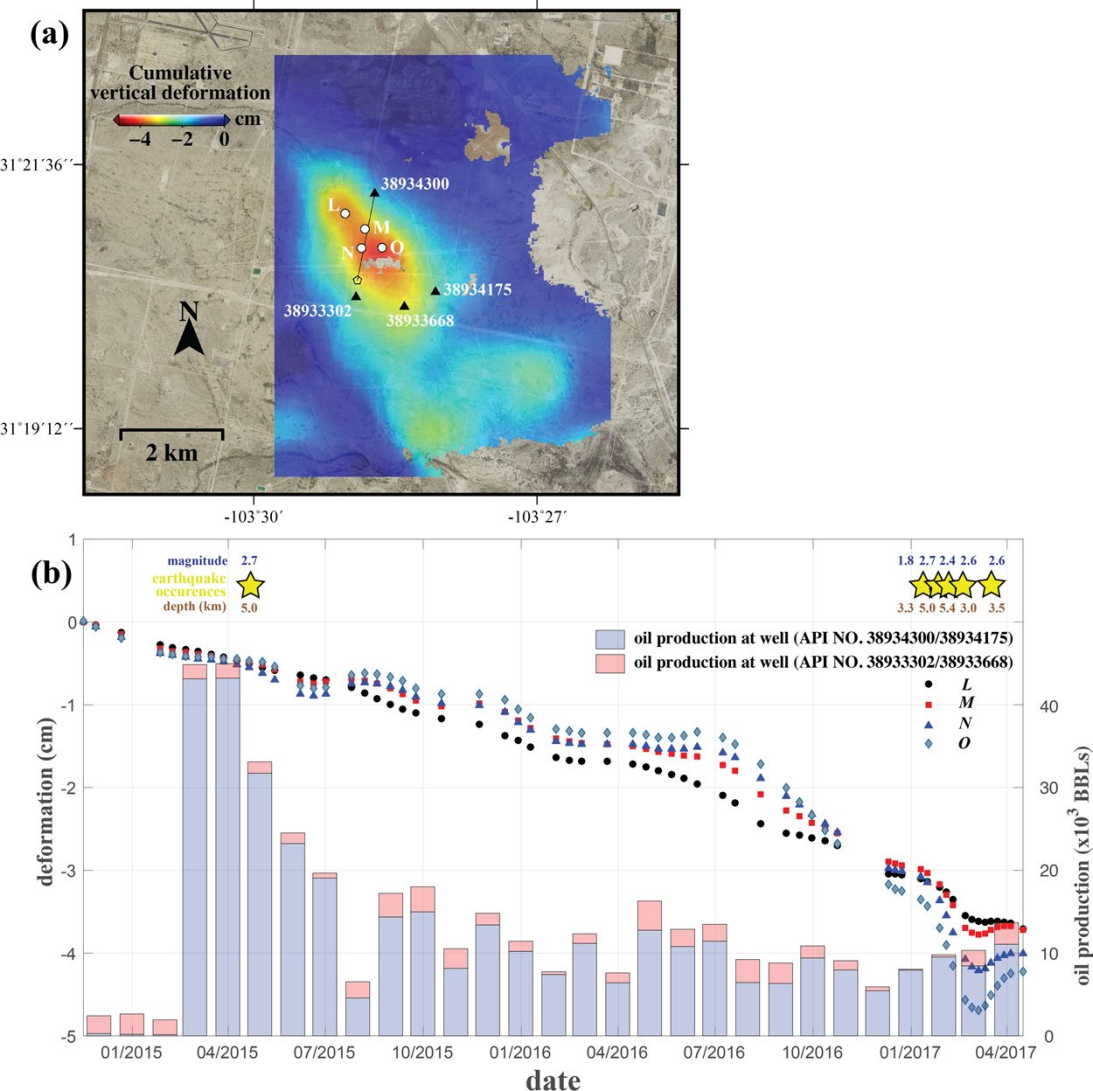
Ground deformation in Pecos, Texas, induced by hydrocarbon production. (**a**) Cumulative ground deformation in a hydrocarbon production field of Pecos, Texas. (**b**) Time-series cumulative vertical deformation in points (L, M, N, O) of Fig. 4a. Yellow stars represent seismic events (along with magnitude and depth) occurring less than 15 km from the subsidence area between late 2014 and April 2017. Oil production (blue and orange bars) volumes in the surrounding wells correlate to the triangles in Fig. 4a. The figure has been created using open-source software GMT 5.2.2_r15292 available at http://gmt.soest.hawaii.edu/projects/gmt/wiki/Download. The NAIP images used as a background of the figures were downloaded in 2017 through Geospatial Data Gateway https://datagateway.nrcs.usda.gov provided by USDA.

The Wolfbone field 9 km south of Pecos, Texas in Reeves County (Fig. 1) has been developed for oil and gas production since 2014. Compared to other oil wells in West Texas that produced hydrocarbon for decades, the wells (API No. 38934300, 38933302, 38933668, 38934175 in Fig. 4a) in the region are recent with significant production exceeding 10,000 BBLs (1 BBL ≈ 0.12 m^3^) starting in early 2015. The drilling depth of the wells is ~4 km below the surface, and most hydrocarbons are produced from the Bone Springs and Wolfcamp formations^47^, which lie in the depth of 2.3~3 km and 3~3.7 km, respectively.

Production from the wells has been enhanced by vertical and horizontal hydraulic fracturing of the sandstone and shale formation. Approximately 4.5 cm subsidence around four producing wells in the Wolfbone field can be observed from our InSAR analysis (Fig. 4a), while the horizontal deformation is negligible. From the time-series measurements in multiple points (Fig. 4b), the subsidence rate has been at a constant, relatively slow speed (1.5 cm/yr) from 2015 to 2016. However, the subsidence was accelerated from January to March 2017 and the amount of the two-month subsidence (O in Fig. 4b) for two months reached up to 1.5 cm (at a rate of ~9 cm/yr). Following the subsidence, the surface uplifted (Fig. 4b) with a maximum magnitude of ~0.5 cm between March and April 2017. We attribute the subsidence to the hydrocarbon production, as most of the subsidence is bounded by production wells in the deep formations^28^ and the extent of the subsidence area is consistent with a source depth of 2–4 km (Fig. 4a). Although the monthly hydrocarbon production exhibits variations, the detected ground subsidence is relatively linear in time. We can postulate that the formations in the subsiding areas behave viscously, different from other observed sites of wastewater injection and CO_2_ flooding. The removal of a huge mass of oil from the subsurface creates the stress changes in the rock/soil layers, but the ground surface gradually responds to such stress changes in the stratigraphy containing abundant viscoelastic shale formations.

Although Pecos, Texas, is located in the geologically stable continental region without any seismic events before 2010s, there have been six small earthquakes in recent years (yellow stars in Fig. 4b). The magnitude of the earthquakes varies between M 1.8 and M 2.7, and all but two events occurred less than 15 km from the subsidence area. Both the timing of the April 2015 earthquake, shortly after the start of the massive increase in oil well production rates, as well as the latest changes in ground surface deformation coinciding the recent five earthquakes in 2017, suggest a close association among ground surface deformation, oil/gas production and seismic events, similar to those observed elsewhere^3,22^. The underlying mechanism to connect oil/and gas production and surface subsidence is that the extraction of oil or gas from underground decreases the pore pressure in formations, which in turn increases the effective stress, which might favor the slip of existing faults.

Hydraulic fracturing along a horizontal pipeline from a horizontal well (such as well No. 38934300) could be responsible for the lateral distribution of vertical displacement in the shale oil field. Moreover, the two-year deformation can accumulate stress on the basement faults near the deforming areas. Although the pre-existing faults in Pecos, Texas have not been documented, the surge in seismic events suggests that faults may exist in the bedrock. Ground subsidence can activate the fault(s)^3,16,48^, and the accelerated subsidence and subsequent uplift in early 2017 can be interpreted as the co-seismic deformation and the viscoelastic relaxation during the short-term post-seismic deformation as a result of the multiple earthquakes^49^. The focal depth of the earthquakes ranges between 3 and 5 km, just slightly below where the hydrocarbon was extracted. Unfortunately, due to the sparse number of seismic stations in Texas, the accuracy in the location of the seismicity can be on the order of 10 km^50,51^. However, the absence of any previously reported regional earthquakes near Pecos, Texas, a shallow focal depth around the producing zone, the proximity of ground deformation to the epicenter therefore suggests a causative link between hydrocarbon production and the sequence of earthquakes (induced seismicity).

## Discussion

**Figure 5:**
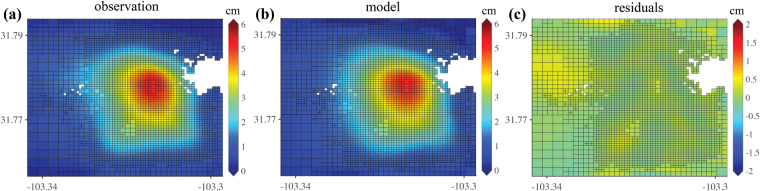
Modeled results of cumulative InSAR vertical deformation around wastewater injection wells. (**a**) observed cumulative vertical deformation in the box outlined by a dashed rectangle (Fig. 2a). (**b**) modeled vertical deformation. (**c**) residuals (observation – model). The figure has been created using MATLAB R2017a licensed by Southern Methodist University.

We have compiled multiple localized geohazards in West Texas (Table 1), most of which were induced by, or at least influenced by, human activities. The correlation between time-series vertical deformation and fluid injection/hydrocarbon production exhibits evident effects of human activities on the surface, but the modeling approach can also help explain the causal relationship. The inverse modeling with the observed cumulative vertical deformation (Fig. 5a) in the box outlined by a dashed rectangle (Fig. 2a) computes the best-fit model (Fig. 5b) with the least residual (Fig. 5c; root mean square (RMSE) misfit: 0.10 cm). The modeled result with a rectangular (3.5 km by 2.5 km) dislocation source at a depth of 1.63 km (known average injection depth) indicates that the peak uplift is located near the wastewater injection well (API No. 49533675). During the observation period of about 2.5 years, both wells (API No. 49533675 and 49530150 in Fig. 2a) had injected saltwater of 5,119,129 BBLs (≈610,408 m^3^) and 3,704,047 BBLs (≈442,623 m^3^), respectively (1,053,031 m^3^ in total). Our computed volume change at the source is about 790,183 ± 8,750 m^3^ and slightly lower than the total injected volume of saltwater. The difference between the two can be attributed to the diffusion of injected saltwater into the surrounding rocks without generating any measurable deformation. The comparable volume change that was calculated from our model reaffirms that the observed surface uplift was induced by disposing a massive volume of saltwater. Although modeling can shed light on revealing the causality of the ongoing ground deformation, we have to realize that models are non-unique and dependent on hydrogeological parameters of the study site. In most general cases where surface uplift and human activities are highly correlated (Fig. 2d), the comparative analysis presented in this report is sufficient for assessing the effect of human activities in West Texas.

**Table 1:** List of the observed ground deformation in West Texas.

Location	Lat/Lon	Basin	Period	Magnitude	Size	Cause	Seismicity
Wink, TX	N31.78° W103.31°	Delaware Basin	01/2016~07/2016	5 cm (uplift)	2.0 × 2.0 km	Wastewater injection	No
Monahans, TX	N31.51° W102.97°	Midland Basin	11/2014~01/2016	3 cm (uplift)	6 × 4 km	CO_2_ injection	No
Grandfalls, TX	N31.27° W102.96°	Delaware Basin	11/2014~04/2017	23 cm (subsidence)	1.4 × 1.0 km	Salt/limestone dissolution	No
Imperial, TX	N31.21° W102.75°	Central Basin Platform	11/2014~04/2017	9 cm/yr (subsidence)	650 × 350 m	Impounded freshwater from abandoned wells	No
Wink, TX	N31.78° W103.12°	Delaware Basin	11/2014~04/2017	40 cm/yr (subsidence)	380 × 280 m	Salt dissolution	No
Pecos, TX	N31.35° W103.48°	Delaware Basin	01/2017~04/2017	4.5 cm (subsidence)	2.5 × 1.0 km	Hydrocarbon production	Yes

Each basin represents the geologic structural basin in the deforming area. Period of the largest gradient of subsidence/uplift, magnitude of maximum cumulative deformation for 2.5 years, and the size of the largest spatial deformation in a region was presented. The cause of deformation was inferred through comparison of the observed deformation, oil/gas production, the volume of the injected fluid, and the geologic characteristics.

Our observations in West Texas can be separated into three groups: i) surface uplift induced by fluid injection, ii) rapid subsidence in a karst terrain due to dissolution of underlying salt deposit, and iii) ground subsidence and seismicity induced by hydrocarbon production. The first category includes two geohazards: one west of Wink, Texas and another southwest of Monahans, Texas. Although both wastewater and CO_2_ injection for EOR are economically efficient to extract oil from reservoirs, the high pressure for raising hydrocarbon production and the increased fluids in the rocks can promote the surface uplift as much as 3~5 cm during an injection. Close correlation between the surface uplift and the injection fluid suggests a causal link between oil producing activities and ground instability.

The second geohazards category includes salt (and perhaps limestone) dissolution in Santa Rosa Spring, Grandfalls and Wink with rapid subsidence in a karst terrain showing the characteristics of a strong linearity regardless of other factors (groundwater, precipitation, temperature, hydrocarbon production). Therefore, the rapid subsidence, once promoted by the freshwater impoundment and the interaction with brine water, is not slowed down by the changes of external effects. In addition, while oil and gas production does not directly impact the subsidence, poor management of the oil and gas facilities, boreholes, and pipelines allows freshwater or brine to interact with Salado salt (and possibly limestone) formations. Their subsidence rate can readily exceed 5 cm/yr, and the subsidence could lead to a collapse at the surface (collapse sinkhole^52^).

Finally, the third geohazards category includes subsidence at the recently developed hydrocarbon sites in Pecos, Texas. Subsidence of ~4 cm in 2.5 years may not be significant on the ground surface, but the continuous subsidence can exert stress on the deep-seated formations and possibly reactivate undocumented faults near the producing zone. West Texas has experienced unprecedented increases of seismicity in last 5–6 years. Earthquakes are occurring in a geologically stable region and the temporal and spatial association with hydrocarbon production suggests that these earthquakes are induced^16^. Based on the accelerated subsidence in 2017, we can hypothesize that the increased number of seismic events is a consequence of the onset of massive hydrocarbon production and thereby ground subsidence after the increase of the effective stress. Contrary to the induced seismicity near the hydrocarbon production in Pecos, Texas, all other ground deformations identified in this study were not followed by seismic events. The ground surface undergoes significant subsidence up to 40 cm/yr (i.e. Wink sinkholes), suggesting that the basement faulting near the producing/deforming zone might not exist and the rapid subsidence can be supported by the underpinning rock formation. Another possibility is that the seismic network in West Texas is neither dense nor sensitive to the micro-earthquakes occurring around the deforming areas^3,9^. In that case, the small-magnitude seismic events could go undetected due to the current sparse placement of seismometers in the area.

Regardless of the occurrence of the induced seismicity, measuring the ground deformation from space in areas where the wastewater and CO_2_ are injected, rocks are dissolving, or the massive hydrocarbons are produced is possible using satellite radar interferometry from new free-data satellites, as we demonstrated in our research. The ground deformation in West Texas is very responsive to anthropogenic activities with little time delay after their implementations were initiated. To avoid more severe geohazards in the future, consideration of such poroelastic movements in producing formations should be carefully heeded.

If we do not mitigate the possible geohazards with continuous monitoring of surface deformation, we can expect one or more possible outcomes: i) damage to infrastructures (roads, railroads, levees, dams), ii) environmental impacts (i.e. ground-water pollution), iii) risks to oil and gas pipelines (note: West Texas has one of the densest networks of oil and gas pipelines in the U.S.), iv) potential threat to residents in surrounding communities, v) economic costs in hydrocarbon productions (i.e. possible improper well managements and thereby ground deformation can lead to large spending by oil companies and governmental agencies to prevent additional damages), and vi) induced seismicity. Micro-seismicity may not result in the large drastic hazards, but the ground deformation (subsidence/uplift) itself poses more direct threat to industrial facilities, infrastructures, and residential areas.

Measuring deformation can assist stakeholders as they examine the safety of the oil and gas operations and make important decisions for securing facilities and people from potential larger catastrophic events. The Texas petroleum regulators have required the submission of historical seismic events in order for an injection/disposal well permit to be approved, but the additional, continuous monitoring of the ground deformation in oil producing areas (regardless of methods including conventional oil production, water flooding, CO_2_ flooding, or hydraulic fracturing) can provide crucial, detailed information for the safe operations of oil and gas productions and the sustainable growth of the energy industry.

## Methods

### Sentinel-1A/B imagery

Sentinel-1A/B, a constellation of two Synthetic Aperture Radar (SAR) satellites operated by the European Space Agency (ESA) within the Copernicus Program, represent the first satellite radar missions providing radar imagery freely to the public. Its radar sensor using interferometric wide swath (IW) mode as a background mode provides C-band (5.4 GHz in center frequency; 5.6 cm in wavelength) imagery with intermediate spatial resolution (20 (azimuth) x 5 (range) m) and dense temporal acquisitions with revisits of 6 days (over Europe, or 12 days outside Europe)^53,54^. For our study, Sentinel-1A/B imagery from November 2014 to April 2017 was been processed. To estimate the deformation in two directions (vertical and east-west), it was necessary to utilize the SAR images from ascending (path 78) and descending (path 85) tracks (Fig. 1), which have the respective heading angle (clockwise from north) of 347.23° and 192.75° with an incidence angle of ~33.8° in the image center. Adaptive multi-look factors were applied to all used SAR images to maintain appropriate spatial resolution in diagnosing the small-sized deformation phenomena.

### Detection of deformation signal and estimation of 2D deformation with InSAR

Because Sentinel-1A/B acquires data with a swath of ~250 km, processing the large size of SAR scene for time-series measurements can be inefficient for detecting a small-sized deformation in West Texas. Moreover, our study area orientation from north to south, required merging of two or more frames. Filtering was applied to interferograms for improving InSAR coherence and also to permit retrieval of the localized deformations in an oil field feasible. To detect numerous deformation signals without losing much spatial resolution, we adopted a stepwise approach of InSAR analysis from a broad to fine scale. In the first run, all available Sentinel-1 images were coregistered based on the SAR image acquired on the first acquisition date. The precise coregistration of Sentinel-1 for avoiding the discontinuous phases between bursts and improving coherence was critical. The process of enhanced spectral diversity (ESD) was iterated until the coregistration precision of azimuth pixel became better than 0.001 pixel^55^, and the pre-resampled SAR images closest to newly-resampled SAR image can aid rapid and more accurate coregistration. All available interferograms with maximum temporal and spatial baselines of 1 year and 200 m, respectively, were generated from the resampled SAR images and were thoroughly examined through visual inspection. Upon discovering the localized signals in our study area, we cropped the interferograms around those deformations. The adaptive multi-look filtering was then applied to each interferogram to maintain high spatial resolution (close to the original resolution of Sentinel-1A/B).

We employed the multi-dimensional small baseline subset (MSBAS) method^56–58^, after removing topographic signatures from interferograms and completing phase unwrapping to estimate the vertical and the horizontal (east-west) deformation from Sentinel-1A/B with two different radar geometries of ascending and descending tracks^59^. Because most SAR sensors are adopting a near-polar orbit and a single (right) look direction, the deformation in north-south direction cannot be resolved without multi-aperture interferometry, along-track interferometry, or offset tracking that is not suitable for mapping small-sized signals. The governing matrix for calculating 2D time-series deformation from multiple tracks is:

1 $$(\begin{array}{ccc}-\frac{4\pi }{\lambda }cos\,\theta \,sin\,\varphi \,A & \frac{4\pi }{\lambda }cos\,\varphi \,A & -\frac{4\pi }{\lambda }\frac{1}{R\,sin\,\varphi }{B}_{p}\\  & \beta I & \end{array})(\begin{array}{c}{V}_{E}\\ {V}_{v}\\ {\rm{\Delta }}h\end{array})=(\begin{array}{c}{\rm{\Phi }}\\ 0\end{array})$$

where *R*, λ, *θ* and *ϕ* are the slant range from the satellite to the target (unit: m), the radar wavelength (~0.056 m), the azimuth angle, and the incidence angle, respectively. When *M*_*k*_ and *N*_*k*_ are the numbers of interferograms and SAR acquisition dates from *k*^*th*^ SAR datasets (assuming that we have *K* (here *K* becomes 2 because we used ascending and descending track) SAR sets), respectively, *A* $({\rm{unit}}:\,{\rm{time}};\,\mathrm{dimension}:\,\sum _{k=1}^{K}{M}_{k}\times (\sum _{k=1}^{K}{N}_{k}-1))$ is a matrix constructed from the time interval between consecutive SAR acquisitions, *β* is a regularization parameter, *I* (dimension: (2$(\sum _{k=1}^{K}{N}_{k}-1)+1))\times (2(\sum _{k=1}^{K}{N}_{k}-1)+1))$ is an identity matrix, *V*_*E*_ and *V*_*v*_ (each has dimensions of $(\sum _{k=1}^{K}{N}_{k}-1)\times 1)$ are the east-west and vertical components (unit: m/time) of the ground deformation rate vector during each time interval, *B*_*p*_ (unit: m; dimension: $\sum _{k=1}^{K}{M}_{k}\times 1$) is the perpendicular baseline, *Δh* is the topography error (unit: m; not significant in a flat region), Φ (dimension: $\sum _{k=1}^{K}{M}_{k}\times 1$) is the observed (unwrapped) interferometric phase (unit: radian), and 0 is a zero vector with a dimension of $(2(\sum _{k=1}^{K}{N}_{k}-1)+1)\times 1$^19,56^ (thus, a left matrix, the unknown vector, and a right vector from (1) have a dimension of $(\sum _{k=1}^{K}{M}_{k}+2(\sum _{k=1}^{K}{N}_{k}-1)+1)$ $\times (2(\sum _{k=1}^{K}{N}_{k}-1)+1))$, ($2(\sum _{k=1}^{K}{N}_{k}-1)+1)\times 1$, and ($\sum _{k=1}^{K}{M}_{k}+2(\sum _{k=1}^{K}{N}_{k}-1)+1)\times 1$). The unknown parameters (*V*_*E*_, *V*_*v*_) were calculated by solving the matrix (1) via singular value decomposition (SVD) with minimum-norm constraints^56^. All used InSAR pairs were connected to each other (full-rank matrix *A*) due to high coherence in our study area, meaning that we have more observations than unknowns (*V*_*E*_, *V*_*v*_, *Δh*). Atmospheric artifacts are not removed separately, because the multi-temporal InSAR using only less-contaminated interferograms could limit the effects of those noises and small areas with ~200–300 m in dimension are less influenced by a large variation of atmosphere. Due to the extreme summer heat in West Texas, the distribution of water vapor in the atmosphere can be still problematic particularly for July and August scenes. However, the spatio-temporal filtering applied to time-series measurements for reducing the effects of water vapor and residual noises worked nicely allowing us to successfully mitigate the influence of those noise and error sources.

However, when the gradient of deformation was large, exceeding 5~10 cm/yr, the interferograms from 24 or more temporal intervals could not maintain coherence. In that case, only the interferograms with 6 or 12-day temporal baselines were used to estimate such a high deformation rate (i.e. rapid subsidence near Wink sinkholes and Imperial, Texas). In West Texas, before mid-2016, most interferograms had 24 day intervals, and most of them were not suitable over the rapidly deforming regions. Therefore, for a large gradient deformation, the limited number of interferograms with short temporal baselines were used by applying a stacking method^60^ that is particularly useful for reducing temporally-uncorrelated signals (atmospheric artifacts, noise) and computing the deformation rate (cm/yr). The peak subsidence near the Wink sinkholes cannot be observed by any 24-day interval Sentinel-1A/B interferograms due to the loss of coherence, but the applied stacking method with 6 or 12-day interval interferograms allows for locating and calculating the maximum subsidence rate in the intersection of County roads 201 and 204.

### Hydrocarbon production and injection volumes

To characterize the ground deformation in West Texas, we performed comparative analysis with information from hydrocarbon production and injection volumes. Records relating to oil/gas production and wastewater injection were collected from the Texas RRC, which is the responsible regulatory authority of the petroleum industry and pipeline safety in Texas. In addition, drillinginfo^TM^ also provided additional information on geological formations in the locations of rapid subsidence. Injection wells, wastewater (generally saltwater) or carbon dioxide (CO_2_) can be injected into an oil-producing unit in the underground for boosting oil and gas production. Most wells used for hydrocarbon production can produce oil and natural gas together, but natural gas, often called casinghead gas, is regarded as a byproduct of these oil wells. Therefore, only the oil production in the wells was considered in our analysis.

### Modeling surface uplift due to wastewater injection

We modeled the cumulative surface uplift to estimate the volume change in the subsurface and assess the relationship between the ground deformation and human activities (here wastewater injection). We used Okada formulation^61^ for motions in a homogeneous elastic half space, because ground deformation presented in our study shows high elastic response to the stress change. The source consists of a planar array of opening cracks at a fixed depth of the wastewater injection. First, we subsampled the cumulative vertical deformation using quadtree downsampling algorithm^62^ for reducing the computational burden in modeling while preserving the statistically significant part of the deformation signal^63^. The best-fitting models were searched over the grid and the best fitting parameters were obtained by minimizing the root mean square (RMS) misfit from the residuals (the observation minus the model)^64,65^.
